# Estimating endogenous changes in task performance from EEG

**DOI:** 10.3389/fnins.2014.00155

**Published:** 2014-06-13

**Authors:** Jon Touryan, Gregory Apker, Brent J. Lance, Scott E. Kerick, Anthony J. Ries, Kaleb McDowell

**Affiliations:** U.S. Army Research Laboratory, Human Research and Engineering DirectorateAberdeen Proving Ground, MD, USA

**Keywords:** EEG, performance estimation, BCI, fatigue, driving, rapid serial visual presentation (RSVP)

## Abstract

Brain wave activity is known to correlate with decrements in behavioral performance as individuals enter states of fatigue, boredom, or low alertness.Many BCI technologies are adversely affected by these changes in user state, limiting their application and constraining their use to relatively short temporal epochs where behavioral performance is likely to be stable. Incorporating a passive BCI that detects when the user is performing poorly at a primary task, and adapts accordingly may prove to increase overall user performance. Here, we explore the potential for extending an established method to generate continuous estimates of behavioral performance from ongoing neural activity; evaluating the extended method by applying it to the original task domain, simulated driving; and generalizing the method by applying it to a BCI-relevant perceptual discrimination task. Specifically, we used EEG log power spectra and sequential forward floating selection (SFFS) to estimate endogenous changes in behavior in both a simulated driving task and a perceptual discrimination task. For the driving task the average correlation coefficient between the actual and estimated lane deviation was 0.37 ± 0.22 (μ ± σ). For the perceptual discrimination task we generated estimates of accuracy, reaction time, and button press duration for each participant. The correlation coefficients between the actual and estimated behavior were similar for these three metrics (accuracy = 0.25 ± 0.37, reaction time = 0.33 ± 0.23, button press duration = 0.36 ± 0.30). These findings illustrate the potential for modeling time-on-task decrements in performance from concurrent measures of neural activity.

## Introduction

Brain-Computer Interaction (BCI) technologies that enable computer systems to adapt to the current cognitive or affective state of the user provide a promising avenue for developing systems that will improve human interaction with computers, the environment, and even each other (Zander and Kothe, 2011; Lance et al., 2012). Among the broad range of BCI technologies, the majority of systems and approaches have been within the active and reactive BCI paradigms (see Zander and Kothe, 2011 for review). These two classes of BCIs seek to decode volitionally induced or externally elicited patterns of neural activity over a relatively short timescale, on the order of milliseconds to seconds. In contrast, passive BCIs utilize implicit or ongoing neural responses for the purpose of detection an operator's current cognitive or affective state. Typically, passive BCIs assess change in neural activity over relatively longer timescales, on the order of seconds to minutes. To date, active and reactive BCI technologies have shown limited success outside of specific patient populations. One reason for this is a lack of robustness, partly due to the non-stationarity of neural signals (Von Bünau et al., 2009; Liyanage et al., 2013). In addition to large inter-session variability, fatigue and other sources of time-on-task decrements in performance can be reflected as a non-stationarity in the neural activity. These effects can be particularly pronounced for tasks that require sustained levels of attention (Tonin et al., 2013). In fact, many active and reactive BCI paradigms are specifically designed to minimize task-induced fatigue and often operate on relatively short timescales (Gao et al., 2014). In this study, we investigate the possibility of addressing this form of non-stationarity through the incorporation of an algorithm designed to identify fatigue-based decrements in performance.

The ability to detect changes in performance directly from biological markers has been an area of growing interest over recent decades. One particularly relevant application is the detection of fatigue, drowsiness, or reduced alertness during driving. Because fatigue is a major cause of accidents and injury when operating motor vehicles (Connor, 2002), robust identification of fatigue before it impairs behavior would be of significant value. To this end, numerous studies have identified indicators of fatigue-induced changes in driver performance from both physiological observables (Vural et al., 2009; Sommer and Golz, 2010; Vogel et al., 2010) and neural signals (Borghini et al., 2012), pre-dominately via electroencephalography (EEG). Furthermore, research groups have recently developed systems for real-time detection of attentional lapses, due to drowsiness or fatigue, from concurrent measures of brain activity (Davidson et al., 2007; Lin et al., 2010a). These approaches fall within the broader passive BCI framework, and are ideal for capturing slow fluctuations in behavioral performance.

Unlike active and reactive BCI paradigms, sustained, and monotonous tasks such as highway driving are used when investigating time-on-task decrements in performance. With such tasks, performance begins to degrade as a function of time, presumably induced by fatigue or inattentiveness due to boredom. Features of the EEG signal, such as fluctuations in power along certain frequencies or changes in evoked amplitudes, can then be correlated with this degradation in performance. Many studies exploring the neural correlates of fatigue use changes in the EEG log power spectrum as principal features in their analysis (Jung et al., 1997; Lal and Craig, 2005; Jap et al., 2009; Balasubramanian et al., 2011). This idea is based on a large body of literature that has linked EEG frequency bands, such as theta (4 to 8 Hz) and alpha (8 to 13 Hz) to changes in task-relevant behavior. In contrast, a more general but potentially powerful approach was originally proposed by Lin et al. (2005a). This approach takes an agnostic view as to the *a priori* selection of frequency bands but rather uses principal component analysis to identify the sets of frequencies that explain the most variance in the EEG power spectrum. The power distribution along these frequencies is then linearly integrated, via a data-driven model, to produce a time-varying estimate of behavior.

While identification of fatigue-induced decrements in driver performance is of obvious importance, other perceptually demanding tasks suffer similar time-on-task decrements, including air-traffic control (Grandjean et al., 1971), and x-ray screening (Basner et al., 2008). Importantly, it is for these types of tasks that the next generation of reactive BCI technology is being developed. However, less is known about the neural correlates of behavior for these more complex tasks. As BCI technologies transition into wider application domains and extended use scenarios, they must be able to adapt to the inevitable fluctuations in human performance. Accordingly, the first step in this process is to understand the link between the neural state and corresponding behavior in the context of time-on-task induced decrements in performance.

Here, we sought to address this important issue by using a data-driven approach to link endogenous changes in behavioral performance to concurrent measures of neural activity. Our goal was to use slow fluctuation in the EEG log power spectrum to estimate time-on-task decrements in performance, based on an extension of a similar BCI paradigm used for drowsiness detection (Lin et al., 2010a), and apply this method to an image triage paradigm increasingly common in BCI technologies (Gerson et al., 2006; Sajda et al., 2010; Touryan et al., 2011, 2013b; Yu et al., 2012). To accomplish this, we designed a study in which participants engaged in both a monotonous driving task and a prolonged rapid serial visual presentation (RSVP) task. Importantly, to quantify the nature and degree of the time-on-task decrements in performance, we acquired subjective, behavioral, and neurophysiologic measures throughout the experiment. We extended the behavior-estimation method found in Lin et al. (2005a), evaluated the method using the data from the simulated driving task, and applied it to the RSVP image triage task. The results of this study suggest that opportunistic identification of time-on-task performance decrements within an event-based BCI is both feasible and advantageous.

## Methods

**Figure 1:**
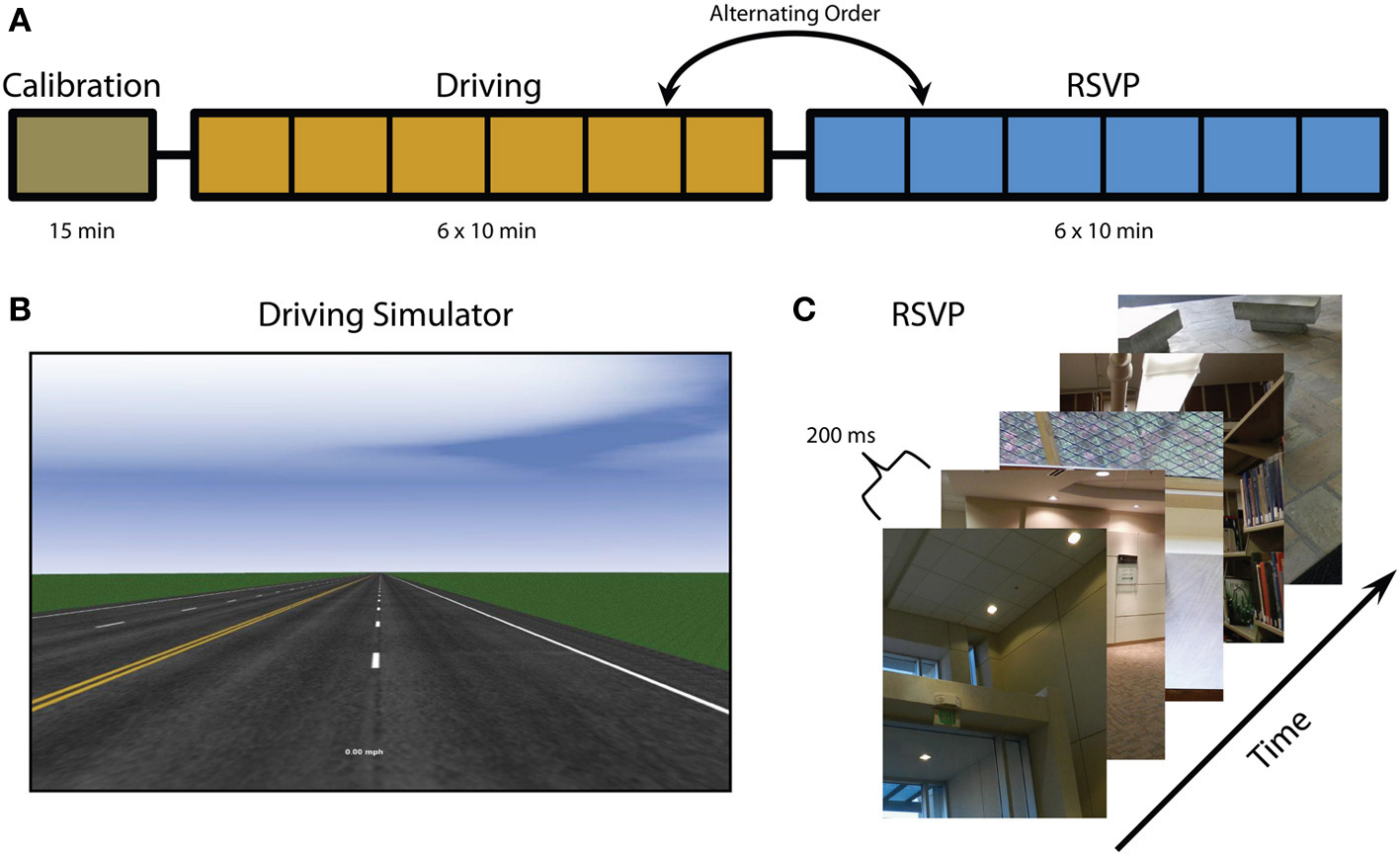
**Experimental overview. (A)** Experiment timeline including calibration, driving, and RSVP tasks (note that the order of the driving and RSVP tasks alternate between participants). Vertical black lines indicate block intervals where fatigue surveys were administered. **(B)** Screenshot of driving simulator. **(C)** RSVP paradigm and example images. Figure adapted from Touryan et al. (2013a).

Twenty-five participants were recruited from the general population. They ranged in age from 21 to 57 (μ = 34.6) and included ten males. Twenty-one of the participants were right handed, two were left handed, and two were ambidextrous. All individuals participated in a single multi-hour session containing three phases and received compensation of $20 per hour. The voluntary, fully informed consent of the persons used in this research was obtained as required by Title 32, Part 219 of the Code of Federal Regulations and Army Regulation 70-25. The investigator adhered to the policies for the protection of human subjects as prescribed in AR 70-25. None of the participants were excluded from the analysis due to noise, movement artifacts, or low behavioral performance. The study design involved 3 tasks (Figure 1): calibration, driving, and rapid serial visual presentation (RSVP). The calibration session was always performed first but the order of the driving and RSVP alternated for each participant.

### Calibration

This task consisted of a standard driving simulator, developed with SimCreator® (Real Time Technologies; Dearborn, MI), that utilized steering wheel and foot pedal controls. In this task the vehicle was moving down a straight highway at a constant speed (computer controlled) in the rightmost lane. Participants were asked to maintain the vehicle position within the current cruising lane by correcting for any perturbation or drift. At random intervals a lateral perturbation to the right or left was applied to the vehicle, causing it to begin to veer off course. The strength of the perturbation increased until a corrective steering adjustment (greater than 4°) was made at which point the perturbation ceased, allowing the participant to return the vehicle to the center of the rightmost lane. The perturbations would only resume once the vehicle was back in the cruising lane for at least 8 s. If the vehicle drifted far beyond the edge of the simulated roadway, participants would receive audible feedback (i.e., rumble strip noise). The simulated environment was minimal and included no traffic or scenery in order to induce boredom and task fatigue. The calibration task consisted of a single 15 min block and was designed for the acquisition of EEG baseline activity.

### Driving

This task was similar to the calibration task except that participants were now given control over the vehicle speed via accelerator and brake pedals. Current vehicle speed was indicated by a digital speedometer at the bottom of the screen. Participants were asked to maintain both the vehicle position and speed. Speed limit signs were posted at regular intervals with values of either 25 or 45 miles per hour. Again, the simulated environment was minimal and included no traffic or scenery. The driving task consisted of 6 blocks of 10 min each with breaks of approximately one minute between blocks.

### RSVP

This task consisted of a rapid presentation of color photographs (512 × 662 pixels) of indoor and outdoor scenes. The images were presented at 5 Hz (200 ms per image) and subtended a visual angle of approximately 9°. Every 10 s a blank screen with the word “blink” was presented to give participants a chance to blink without missing stimuli. The RSVP task consisted of 6 blocks of 10 min each (to mirror the driving task). All scenes contained only inanimate objects and were manually scaled and cropped. Some scenes contained target objects and others did not. Before each block participants were instructed as to the class of target objects for that block. The target classes for this experiment were: stair, container, poster, chair, and door. Before the task began, participants were familiarized with exemplars from each target class. During the RSVP, participants were instructed to press a button only when they saw an object from the current target class. The order of the target classes was randomly chosen for each participant (blocks 1–5); however, the last block (block six) always had the same target class as the first block. In addition to target class, target probability varied across each block. Six target probability values (0.01, 0.03, 0.05, 0.07, 0.09, and 0.11), one for each block, were randomly assigned at the beginning of the task.

#### Subjective measures

In addition to biographical information, various cognitive and personality metrics were obtained, via standard questionnaires or timed assessments at the beginning of the experiment. The data from these cognitive and personality assessments was not included in the present study. Self-reports of fatigue were obtained using three different questionnaires: (i) the Visual Analog Scale for Fatigue (VAS-F; Monk, 1989), (ii) the Task-Induced Fatigue Scale (TIFS; Matthews and Desmond, 1998), and (iii) the Karolinska Sleepiness Scale (KSS; Akerstedt and Gillberg, 1990). The VAS-F was administered once after each task (calibration, driving and RSVP). The TIFS and KSS were administered once after the calibration task, after each 10 min block in the driving and RSVP tasks, and once at the end of the experiment. In order to account for individual differences in basal fatigue level, scores were normalized by the mean value over the experiment for each participant.

#### Behavioral measures

During the driving simulator task, various vehicle state measures were acquired at 100 Hz. Since the task objective was to maintain vehicle position within the rightmost lane, lane deviation (the difference between the vehicle's lateral position and the center of the lane) was the metric used to assess driver performance. During the RSVP task, participants pressed a button only when they saw a target object. Accuracy, reaction time (RT) and press duration were determined from this button response. Because the image duration (200 ms) was much less than the average RT, button responses were assigned to images in the following manner. For each button press, images within the time window of 300 to 1000 ms preceding the response were identified. If one or more of these preceding images was a target, the button press was assigned to the first (oldest) target image. RT was then calculated from the onset of that target image. If no targets occurred within the preceding time window, the button press was assigned to the non-target image that preceded the button press by 600 ms (a standard RT value). However, due to the ambiguity of assigning a button press to a non-target image, RT statistics were not calculated for non-target images and this assignment process was only used to determine the false alarm rate.

For the RSVP task, behavioral responses were strongly influenced by perceptual difficulty; some targets were obvious and identified in all instances while other targets were subtle and only identified in some instances. The effect of perceptual difficulty was even evident at the aggregate level of target class (see Table 3), where the average accuracy was greater for some classes of target objects (e.g., chair) relative to others (e.g., poster). Therefore, to mitigate the influence of perceptual difficulty, we calculated a normalized behavioral metric. Specifically, average accuracy, RT, and duration were calculated for each target image across all participants (grand average). Then, for each instance of that target within the RSVP stream this grand average was subtracted from the behavioral response. This difference was then added to the nominal value for each measure. For accuracy, the nominal value was one: accuracy values greater than one indicated the participant was more accurate than average for that target image. For RT, the nominal value was 600 ms: RT values greater than 600 ms indicated the participant was slower than average for that target image. Lastly, the nominal value for duration was 300 ms: duration values greater than 300 ms indicated the participant depressed the button longer than average for that target image. These nominal values were chosen as round numbers that reflect the average behavioral response across participants. In addition to perceptual difficulty, target probability had a pronounced effect on RT and button press duration. Across participants, both RT and duration decreased as target probability increased (RT: slope = −262 ms, *p* < 0.05; duration: slope = −277 ms, *p* < 0.05). Using the inverse of these slopes, we adjusted both RT and duration as a function of target probability, on a block-by-block basis.

To capture temporal fluctuations in performance during both driving and RSVP tasks, we averaged the behavioral metrics (driving: absolute lane deviation, RSVP: accuracy, RT, and duration) via a centered, 90 s mean filter (Jung et al., 1997; Lin et al., 2005b; Chuang et al., 2012). This window size provided a robust, time-varying estimate of accuracy, RT and duration (average number of trials per window = 445) even when the target probability was low. The filtered data were center-aligned such that each time point included an average of data over the preceding and following 45 s. The edges of the filtered data were padded with the first and last valid value after smoothing (i.e., 45 s after the beginning of the first block and 45 s before the end of the last block).

#### Electroencephalography measures

Electrophysiological recordings were digitally sampled at 1024 Hz from 256 scalp electrodes over the entire cortex using a BioSemi Active Two system (Amsterdam, Netherlands). External leads were placed on the outer canthus, and above and below the orbital fossa of the right eye to record electrooculography (EOG). For power spectrum analysis, EEG was referenced to the average mastoids, down-sampled to 256 Hz, and digitally band-pass filtered between 0.5 and 50 Hz using the EEGLAB toolbox (Delorme and Makeig, 2004). A down-selected montage was created by (i) identifying the subset of channels that matched the BioSemi 32 configuration and, (ii) averaging the electrodes directly adjacent to those channels (between 5 and 8 depending on location). The purpose of the neighborhood averaging was merely to mitigate the influence of noise or high impedance from a single channel. In our approach, spatial filtering was accomplished through principal component analysis (PCA, see below) rather than the application of spherical Laplacian or identification of the independent component.

**Figure 2:**
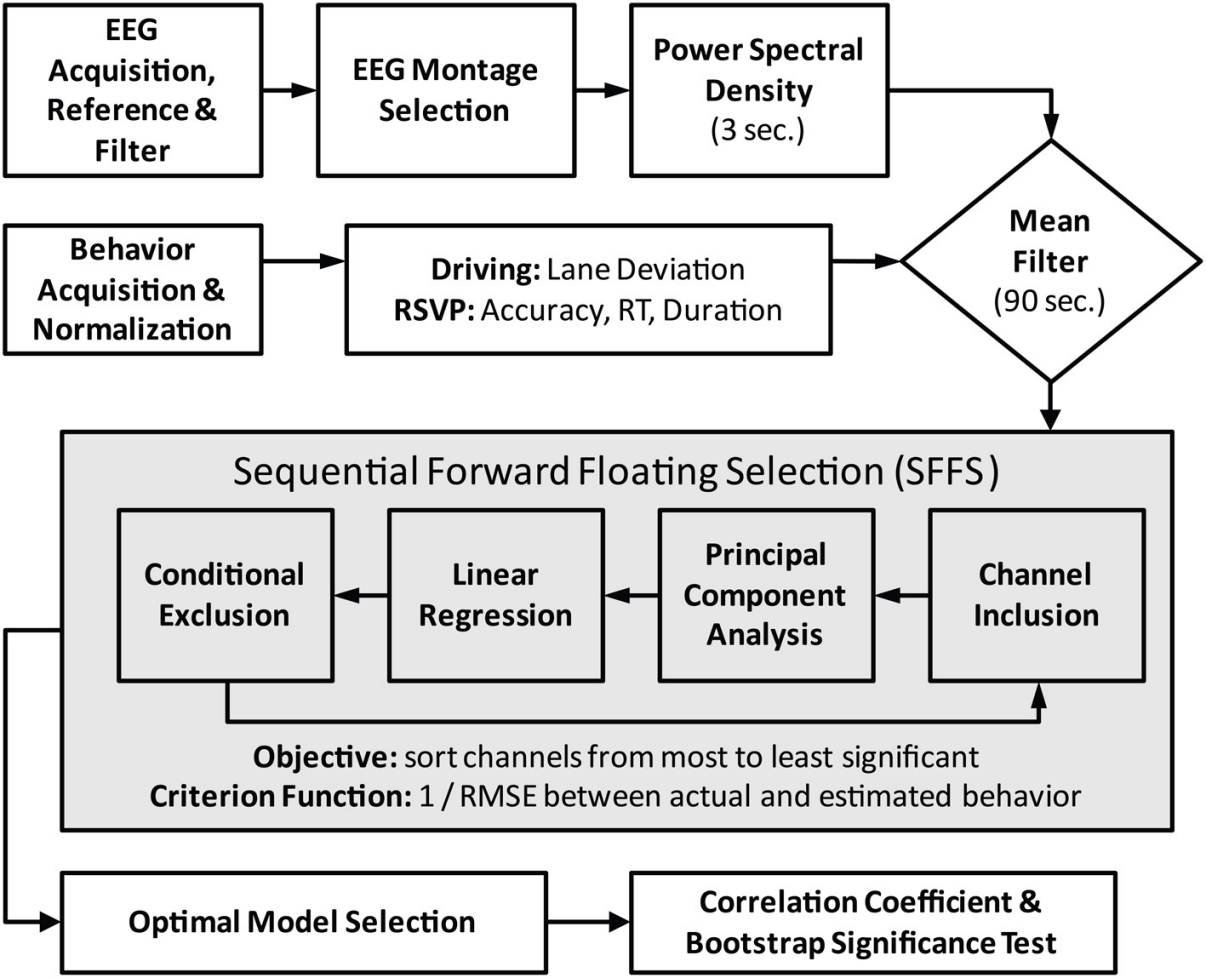
**Flowchart of data preprocessing and model fitting**. Central box encapsulates the iterative process of feature (channel) selection via the SFFS algorithm. For the standard modeling scheme the feature selection component is replaced by the fixed midline montage: Fz, Cz, Pz, and Oz.

Moving-average power spectra were based on an approach described by Lin et al. (2005a). Briefly, the power spectral density (PSD) estimates were calculated in sliding 750-point epochs (~3 s) with a 500-point step size (~2 s). Each epoch was subdivided into 125-point Hanning windows with a 25-point step size. A 256-point FFT was then used to calculate the power spectrum for each window and a 5th order median filter was applied across windows for artifact mitigation. The windowed spectra were then averaged and converted into a logarithmic scale to produce the time-varying PSD estimate for each channel. Frequencies between 1 and 40 Hz were kept for subsequent analysis. Finally, the power estimates at these frequencies were smoothed with a 90 s mean filter in the identical fashion as the behavioral metrics described above. Figure 2 outlines the sequence of steps in the EEG preprocessing, behavior integration, and model building components of the analysis.

#### Regression models

Two modeling schemes were used for each participant and task, a standard modeling scheme and an adaptive modeling scheme. In the standard scheme, regression models were built with the PSD estimates from four midline electrodes: Fz, Cz, Pz, and Oz. PSD estimates from these channels were combined to form a high-dimensional vector of the EEG log power spectrum.

(1) $$\;X_{4}=\left(\begin{array}{ccc} {\left(Fz_{1}\ldots Fz_{40}\right)}_{1} & \cdots & {\left(Oz_{1}\ldots Oz_{40}\right)}_{1}\\ \vdots & \ddots & \vdots \\ {\left(Fz_{1}\ldots Fz_{40}\right)}_{n} & \cdots & {\left(Oz_{1}\ldots Oz_{40}\right)}_{n} \end{array}\right)$$

Here, *X*_4_ is the matrix of combined PSD estimates from the 4 channels and *n* overlapping time epochs. PCA was then applied to the combined PSD estimates (2). The set of eigenvectors *V* that explained at least 1% of the variance were then selected to represent the subspace of EEG log power (3),

(2) $$C_{X}\;=V\left(\begin{array}{ccc} \lambda_{1} & \cdots & 0\\ \vdots & \ddots & \vdots \\ 0 & \cdots & \lambda_{160} \end{array}\right)V^{-1}$$

(3) $$V=\left\{v_{i}|\frac{\lambda_{i}}{\sum \lambda }\geq 0.01\right\}$$

where *C_X_* is the covariance matrix of the combined PSD estimates over the experiment (*X*_4_) and *v_i_* and λ_*i*_ correspond to the *i*th eigenvector and eigenvalue respectively. A linear regression model, with a least-squares-error cost function, was fit to the behavioral data using the PSD projections onto these eigenvectors. No explicit temporal offset or lag is included in the regression model as the eigenvectors represent only a single PSD epoch.

For the adaptive modeling scheme, regression models were built using a subset of electrodes selected from the entire 32 channel montage. A different number and subset of channels was used for each participant and task to maximize the model's performance. Specifically, sequential forward floating selection (SFFS) was utilized to rank channels in order of significance (Pudil et al., 1994). An iterative process added and removed channels from the rank-ordering by maximizing the criterion function *J*(*X_k_*) at each step (Table 1). The criterion function, one over the root-mean-squared error, was calculated as follows:

(4) $$J\text{​}\left(X_{k}\right)=\frac{1}{{\left(\frac{1}{n}\sum_{i=1}^{n}{\left(y-y_{est}\text{​}\left(X_{k}\right)\right)}^{2}\right)}^{1/2}}$$

where *X_k_* is the combined PSD from *k* channels, *y* is the actual behavior, and *y_est_* (*X_k_*) is the estimated behavior using these channels. During each iteration, PSD estimates from *k* channels were combined to form a high-dimensional vector of EEG log power. PCA was then applied to the combined PSD estimates. As in the standard scheme, eigenvectors that explained at least 1% of the variance were then selected to represent the subspace of EEG log power. A linear regression, with a least-squares-error cost function, was fit to the behavioral data using the PSD projections onto these eigenvectors.

**Table 1:** **Sequential forward floating selection (SFFS) algorithm**.

**Step**	**Operation**
1.	Let *k* = 0, *X_k_* = {∅}
2.	Select next most significant feature:
	*x*_*k* + 1_ = max_*x* ∉ *X_k_*_ *J* (*X_k_* + *x*)
	*X*_*k* + 1_ = *X_k_* + *x*_*k* + 1_
3.	If *J* (*X*_*k* + 1_ − *x*_*k* + 1_) ≥ *J* (*X*_*k* + 1_ − *x_j_*), ∀*j* = 1, 2,… *k*, then
	*k* = *k* + 1
	go to step 2
	Else, exclude least significant feature from *X*_*k* + 1_
	*x_r_* = arg max_*x* ∈ *X_k_*_ *J* (*X_k_* − *x*)
	*X*′_*k*_ = *X*_*k* + 1_ − *x_r_*
4.	Find least significant feature in *X*′_*k*_:
	*x_s_* = arg max_*x* ∈ *X*′_*k*__ *J* (*X*′_*k*_ − *x*)
5.	If *J* (*X*′_*k*_ − *x_s_*) ≤ *J*(*X*_*k* − 1_), then
	*X_k_* = *X*′_*k*_
	go to step 2
	Else, exclude least significant feature from *X*′_*k*_
	*X*′_*k* − 1_ = *X*′_*k*_ − *x*_*s*_
	*k* = *k* − 1
	repeat steps 4 and 5

By iteratively including and excluding channels, the SFFS algorithm avoids local maxima and can therefore be used to find the globally optimal feature set. For this dataset, the criterion function tended to peak well before all channels were included in the rank-ordering. Therefore, to reduce computational time we included a maximum-iteration number of 500 for our SFFS implementation. With this value, the criterion function for each participant achieved its peak value and an increase in iteration number did not improve performance (data not shown). The final behavioral estimate was generated using the set of channels with the largest *J* (*X_k_*). For the current study, *k* ranged between 1 and 12 channels.

For both the standard and adaptive modeling schemes, the EEG and behavioral data were split into leave-one-out cross-validation sets corresponding to the experimental blocks. Specifically, models were built with data from five blocks and tested on data from the remaining block. This cross-validation procedure insured a temporal separation between the training and testing sets roughly the size of the 90 s smoothing window (see Supplementary Material). The model performance was quantified using Pearson's correlation coefficient between the actual (*y*) and estimated (*y_est_*) behavior.

(5) $$R=\frac{\sum \text{​}\left(y-\bar{y}\right)*\left(y_{est}-\bar{y_{est}}\right)}{\sqrt{\sum \text{​}{\left(y-\bar{y}\right)}^{2}*\sum \text{​}{\left(y_{est}-\bar{y_{est}}\right)}^{2}}}$$

Here, significance was established using a bootstrap reshuffling technique. Specifically, values of the estimated behavior vector (*y_est_*) were randomly permuted and then smoothed by a 90 s mean filter. The correlation coefficient between the random estimate and the actual behavior was then calculated. The correlation coefficients from 1000 permutations were used to estimate the mean and variance of the random distribution for each behavior vector and establish a significance threshold (*p* < 0.05).

To determine the spectral characteristics of the regression model, we calculated the relative contribution of each frequency component (1 to 40 Hz) to the overall behavioral estimate. Specifically, the relative weight for each frequency would be calculated as follows:

(6) $$W(f)=\frac{1}{\left\|\beta \right\|}\sum_{i=1}^{M}\hat{X}(f)v_{i}(f)\beta_{i}$$

Here, $\hat{X}(f)$ and *v_i_*(*f*) are the value of the average PSD and *i*th eigenvector at frequency *f*, respectively. The average PSD and eigenvector are weighted by the corresponding linear model coefficient β_*i*_. The resulting sum is normalized by the magnitude of the model coefficients to compare across participants. Relative weights were calculated for all *k* channels and averaged across training sets. We used the Benjamini and Hochberg (1995) false discovery rate (FDR) algorithm to determine which frequencies had relative weights significantly different from zero (*p* < 0.05) across all channels and participants.

## Results

### Subjective measures

**Figure 3:**
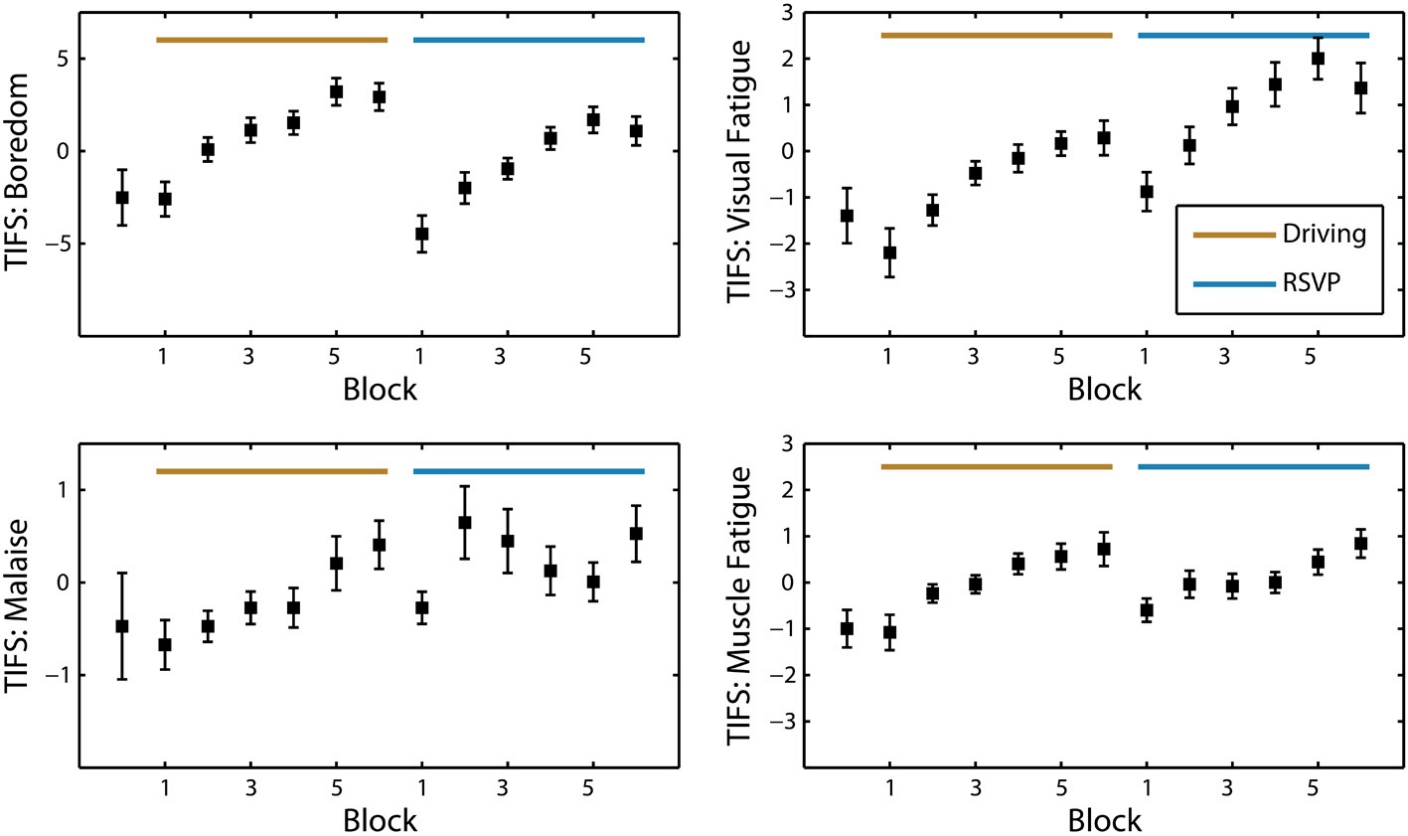
**Average TIFS scores across participants (*N* = 25)**. Grand average of the TIFS scores with standard error at every block interval. The four dimensions of TIFS are represented (boredom, visual fatigue, malaise, and muscle fatigue). Scores are normalized by the mean of each participant and sorted for alternating task order.

Over the population, self-reported fatigue increased during both the driving and RSVP tasks (Figure 3). To assess the significance of this trend we performed repeated-measures ANOVA for each task type and survey with block or interval as the main factor (see Table 2). The Karolinska Sleepiness Scale (KSS) showed significant time-on-task effects in the driving and RSVP portion of the experiment (*p* < 0.001). Similarly, the Task-Induced Fatigue Scale (TIFS) showed time-on-task effects along 3 of the 4 the dimensions (*p* < 0.001); this included boredom, visual fatigue, and muscle fatigue. Malaise showed a significant time-on-task effect for only the driving portion of the experiment (*p* < 0.01). The TIFS also revealed a significant task type effect for boredom and visual fatigue (*p* < 0.01), where the driving task was perceived as more boring, and the RSVP task was perceived as inducing more visual fatigue.

**Table 2:** **ANOVA for KSS, VAS-F, and TIFS**.

**Factor**	***df***	***F***	***p***
**KSS**			
Driving block	5,24	5.21	<0.001
RSVP block	5,24	7.72	<0.001
Task type	1,24	0.01	0.906
**VAS-F**			
Interval	2,24	1.36	0.267
**TIFS BOREDOM**			
Driving block	5,24	11.80	<0.001
RSVP block	5,24	12.15	<0.001
Task type	1,24	11.57	0.001
**TIFS VISUAL FATIGUE**			
Driving block	5,24	9.05	<0.001
RSVP block	5,24	5.98	<0.001
Task type	1,24	30.49	<0.001
**TIFS MALAISE**			
Driving block	5,24	3.65	0.004
RSVP block	5,24	1.69	0.142
Task type	1,24	7.14	0.008
**TIFS MUSCLE FATIGUE**			
Driving block	5,24	6.95	<0.001
RSVP block	5,24	6.04	<0.001
Task type	1,24	0.05	0.820

Not surprisingly, many of the subjective measures seemed to plateau or decrease prior to the last block of the task. In the beginning of the experiment, participants were informed how many blocks would be included in each task. Thus, individuals seemed to experience an increased alertness as they neared the end of the task, a phenomenon shown in previous studies (Lorist et al., 2009). In contrast to the KSS and TIFS, the Visual Analog Scale for Fatigue did not show a significant time-on-task effect. However, this survey was only administered three times during the entire experiment (at the beginning, once after the driving task, and once after the RSVP task). Overall, the participant reports of fatigue indicated that both the driving and RSVP task induced fatigue and boredom.

### Behavioral measures

A number of previous studies have sought to quantify driver performance with a range of metrics including lane position, reaction time to perturbation onset, and corrective steering wheel deflections, among others. For simplicity we used absolute lane deviation as a general proxy for driver performance and level of alertness (Sandberg et al., 2011). Across participants, there was no significant increase in either the mean or standard deviation of absolute lane deviation across blocks. However, we did observe that lane deviation had a tendency to increase throughout each block, returning back to a lower value at the beginning of the subsequent block (Figure 6A). To quantify this effect, we fit a linear function to the absolute lane deviation within each block. We found that this slope exhibited a time-on-task effect [*F*_(5, 24)_ = 2.42, *p* < 0.05], with sharper increases in lane deviation during later experimental blocks.

**Figure 4:**
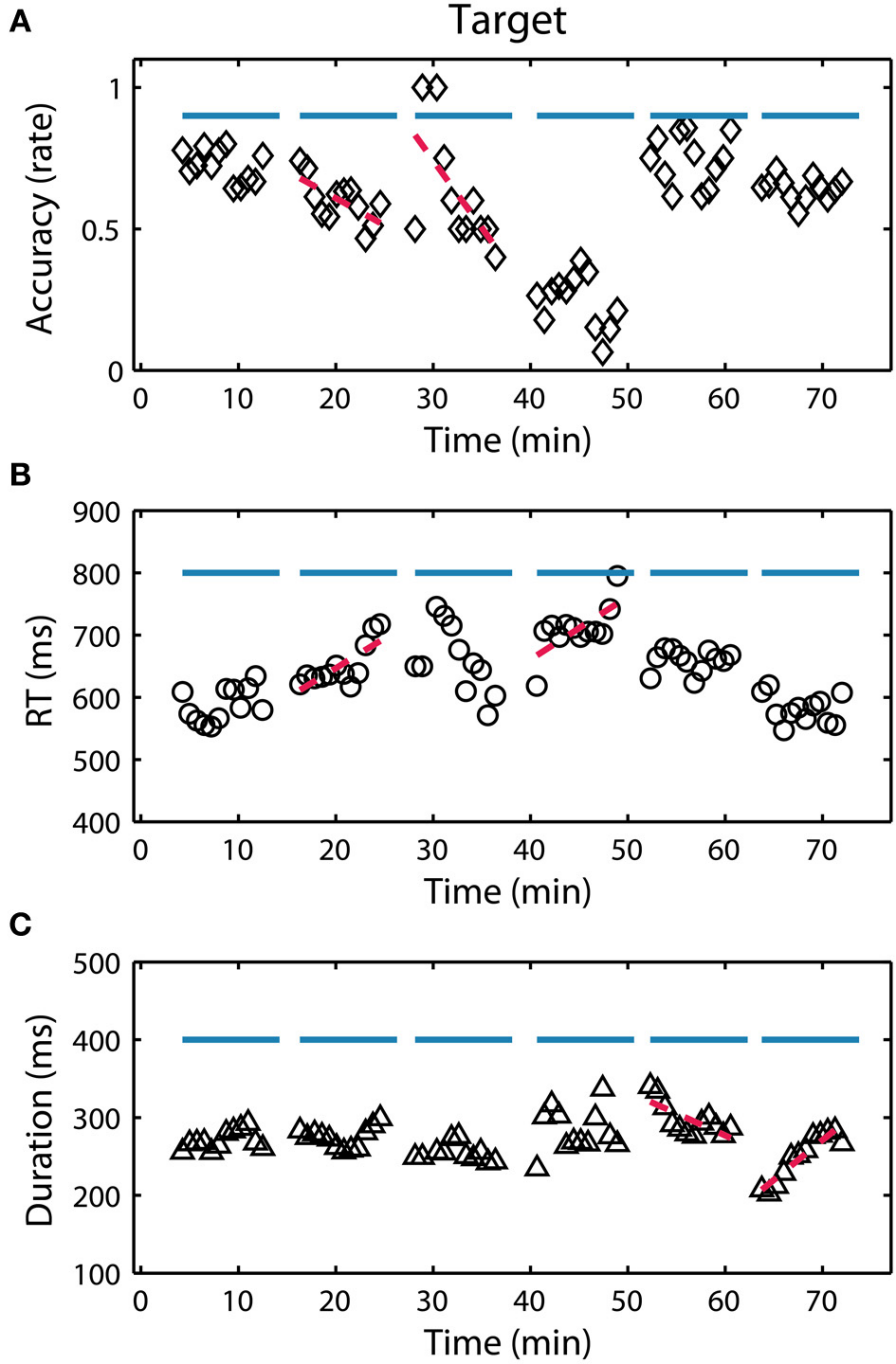
**RSVP behavior for participant S08. (A)** Target detection accuracy (hit rate) over the 6 experimental blocks (indicated by horizontal blue lines). Each point represents a calculation of the metric over a 90 s integration window representing approximately 445 trials (target and non-target). Red lines indicate a significant (*p* < 0.05) linear relationship between the behavior and time within the block. **(B,C)** Show the reaction time (RT) and button duration over the same period.

Figure 4 shows the temporal dynamics of the three RSVP behavioral metrics (accuracy, RT and button press duration) for a typical participant. Notably, there were large fluctuations both within and across blocks. Across blocks, these fluctuations could be due to changes in either task parameters (target class or target probability) or alertness level. Within block fluctuations, however, could only be precipitated from endogenous changes such as perceptual learning, fatigue, or boredom. To further quantify these fluctuations we calculated both the average behavioral performance across each block and identified significant linear trends within each block.

First, to assess the influence of the task parameters we performed separate repeated-measures ANOVAs on accuracy, RT and button press duration with block, target class, and target frequency as factors (see Table 3). Across participants, one clear modulator of performance in the RSVP task was target class. This was true for both accuracy and RT (*p* < 0.001 for both), and to a lesser extent button press duration (*p* < 0.05). Likewise, target probability had a significant effect on RT and duration (*p* < 0.001) but not accuracy. Although none of the raw behavioral metrics showed a significant time-on-task effect across blocks, most participants had at least one block with a significant decrease in accuracy or increase in RT, reflecting a within block time-on-task performance decrement. To quantify this, for each participant we identified all blocks in which the accuracy or RT had a significant linear trend (*p* < 0.05). Time-on-task performance decrements were defined as blocks with either significantly negative trends in accuracy or significantly positive trends in RT. On average, participants exhibited this type of performance decrement in multiple blocks (μ = 2.12 blocks per participant). Similarly, performance improvements were defined as blocks with either significantly positive trends in accuracy or significantly negative trends in RT. In contrast to decrements, participants exhibited these performance improvements far less often (μ = 0.52 blocks per participant). This difference was significant across participants (*p* < 0.001; paired *t*-test), indicating that fatigue or boredom had a more pronounced influence on within block performance as compared with perceptual learning.

**Table 3:** **ANOVA for behavioral measures in the RSVP task**.

**Factor**	**Raw**			**Normalized**		
	***df***	***F***	***p***	***df***	***F***	***p***
**ACCURACY**						
Block	5,24	1.63	0.157	5,24	4.26	0.001
Target class	4,24	31.17	<0.001	4,24	1.06	0.379
Target frequency	1,24	0.00	0.950	1,24	0.48	0.492
**REACTION TIME**						
Block	5,24	1.57	0.173	5,24	1.28	0.279
Target class	4,24	9.09	<0.001	4,24	2.38	0.056
Target frequency	1,24	16.77	<0.001	1,24	0.00	0.953
**BUTTON PRESS DURATION**						
Block	5,24	2.18	0.061	5,24	1.73	0.132
Target class	4,24	2.54	0.043	4,24	1.40	0.238
Target frequency	1,24	29.43	<0.001	1,24	0.09	0.766

**Figure 5:**
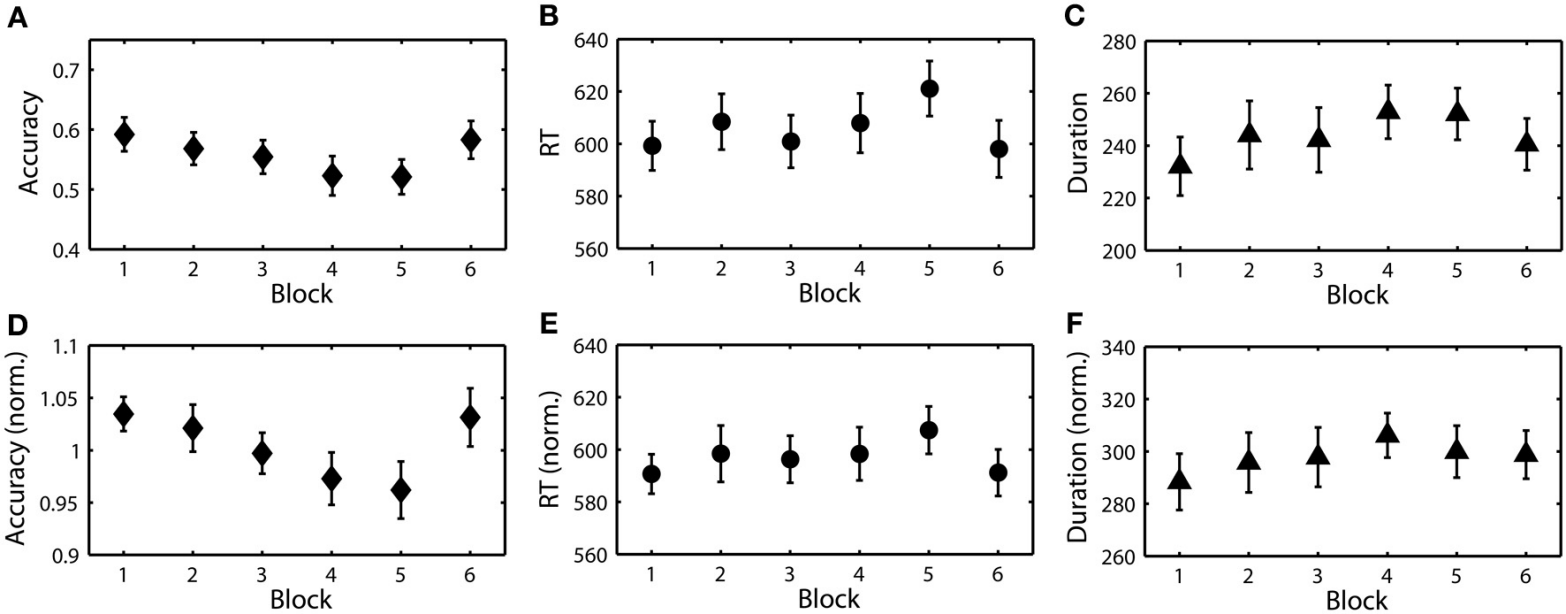
**Raw and normalized RSVP behavioral measures. (A)** Grand average target detection accuracy over blocks. **(B)** Grand average RT (ms). **(C)** Grand average button press duration (ms). **(D–F)** Normalized accuracy, RT, and duration over the same blocks.

Since the goal of this study was to identify endogenous changes in performance, particularly task-induced fatigue or boredom, we wanted to mitigate the influence of task parameters on behavior. To accomplish this we normalized the behavior (accuracy, RT, and duration) for perceptual difficulty and target probability. Figure 5 shows the results of this normalization process for all three behavioral metrics. While a subtle time-on-task trend is evident in the raw accuracy, it is masked by the effect of target class. In contrast, with normalized accuracy the time-on-task trend becomes highly significant (see Table 3). Interestingly, normalized RT and duration did not exhibit a similar time-on-task effect across blocks. Using these normalized metrics, we then developed EEG-based models of the RSVP behavior for each participant.

### Estimating performance from EEG

**Figure 6:**
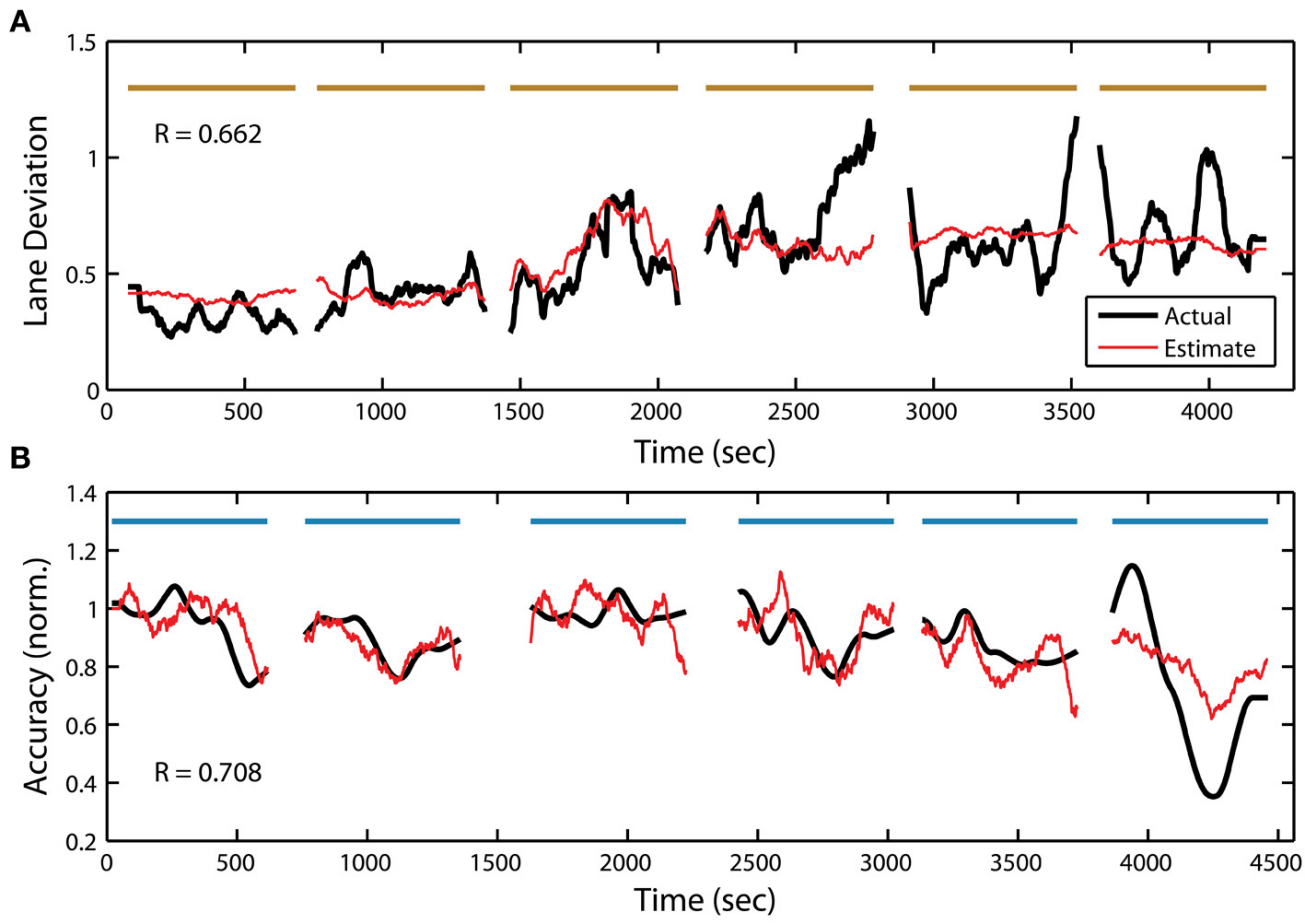
**Continuous measure of driving and RSVP (target detection accuracy) behavior for participant S22. (A)** Actual and estimated absolute lane deviation (meters) over the 6 driving blocks. **(B)** Actual and estimated normalized accuracy over the 6 RSVP blocks. Horizontal bars indicate experiment blocks.

Previous studies have shown a clear relationship between the EEG power spectrum and time-on-task decrements in performance, especially in monotonous driving (Ting et al., 2008) or vigilance tasks (Stikic et al., 2011). Less is known about the link between the EEG power spectrum and behavior in perceptual tasks, such as the RSVP paradigm described here. To explore this relationship further, we constructed linear regression models to estimate each participant's behavior from their EEG power spectral density (PSD). A separate set of linear models were created from the PSD for both the driving and RSVP tasks using an adaptive modeling scheme. Figure 6A shows the actual and estimated behavior (absolute lane deviation) for one participant in the driving task. Notably, there was substantial variability in model performance across blocks. This indicated that the underlying relationship between the PSD and driving performance was variable between training and testing sets (Apker et al., 2013). Figure 6B shows the actual and estimated behavior (normalized accuracy) for one participant in the RSVP task. As with the regression models of driving behavior, these models show some degree of variability across blocks.

To quantify the accuracy of these estimates we calculated Pearson's correlation coefficient, between the actual and estimated behavior, over the entire task. In line with our previous studies (Touryan et al., 2013a), we found that we were able to produce a behavioral estimate with a significant correlation coefficient for the majority of participants (see Table 4). This was true for both the driving (21 of 25 participants) and RSVP tasks (14 of 25 participants). Across participants, there was no significant difference in the accuracy of the behavioral estimate in the two tasks (*R* = μ ± σ; driving *R* = 0.374 ± 0.224, RSVP *R* = 0.248 ± 0.368, *p* = 0.14; Wilcoxon signed-rank test). In contrast, these results were substantially better than could be achieved using a standard, fixed-montage approach. Specifically, the standard modeling scheme only yielded significant estimates for 6 participants in the driving task and 4 participants in the RSVP task. The average accuracy of the behavioral estimate was also significantly lower than the adaptive approach for both the driving (*R* = 0.079 ± 0.224, *p* < 0.001) and RSVP (*R* = −0.137 ± 0.332, *p* < 0.001) tasks.

**Table 4:** **Regression model performance for the driving and RSVP tasks**.

**Participant**	**Driving**		**RSVP-accuracy**		**RSVP-RT**		**RSVP-duration**	
	**RMSE^a^**	***R***	**RMSE^a^**	***R***	**RMSE^a^**	***R***	**RMSE^a^**	***R***
1	0.78	0.63^***^	0.92	0.40^**^	0.92	0.40^**^	0.98	0.30^*^
2	1.00	0.21^*^	0.81	0.59^***^	1.13	−0.11	1.09	−0.03
3	0.85	0.54^***^	0.84	0.54^***^	1.01	0.17	0.95	0.36^**^
4	1.21	−0.22	0.88	0.48^***^	0.82	0.58^***^	0.87	0.52^***^
5	0.97	0.31^**^	0.99	0.20	0.98	0.36^**^	0.99	0.24
6	1.04	−0.03	0.58	0.82^***^	0.77	0.64^***^	0.81	0.58^***^
7	0.88	0.48^***^	1.40	−0.80	1.08	0.06	1.09	−0.06
8	0.95	0.32^***^	1.00	0.30^*^	0.81	0.60^***^	1.01	0.16
9	1.03	0.17	1.05	0.13	1.02	0.10	1.00	0.26^*^
10	0.81	0.60^***^	0.93	0.40^**^	1.01	0.19	1.08	−0.03
11	0.86	0.51^***^	1.01	0.28^*^	0.93	0.39^**^	0.73	0.68^***^
12	0.96	0.32^**^	0.98	0.20	0.91	0.43^**^	0.85	0.52^***^
13	1.07	−0.08	1.03	0.15	0.83	0.57^***^	0.78	0.63^***^
14	0.86	0.52^***^	0.97	0.31^*^	1.05	0.16	1.03	0.17
15	0.91	0.42^***^	0.97	0.30^*^	0.95	0.33^*^	0.94	0.35^**^
16	0.91	0.44^***^	0.75	0.66^***^	0.73	0.70^***^	0.98	0.34^**^
17	0.91	0.43^***^	1.01	0.18	1.07	0.09	0.92	0.46^**^
18	0.92	0.43^***^	1.00	0.28^*^	1.05	0.24	0.70	0.72^***^
19	0.90	0.53^***^	1.31	−0.12	0.83	0.58^***^	1.17	−0.01
20	0.75	0.66^***^	0.71	0.71^***^	0.94	0.41^**^	0.80	0.61^***^
21	1.11	0.37^**^	0.81	0.59^***^	1.05	0.16	0.77	0.64^***^
22	0.78	0.64^***^	1.01	0.14	0.90	0.48^***^	0.40	0.92^***^
23	0.96	0.31^**^	1.01	0.23	0.80	0.60^***^	0.90	0.45^**^
24	0.89	0.46^***^	1.21	−0.26	1.15	0.09	1.27	−0.37
25	0.92	0.39^***^	1.30	−0.52	1.03	0.09	0.81	0.59^***^
Average	0.93	0.37	0.98	0.25	0.95	0.33	0.92	0.36

a Root-mean-squared error (RMSE) values have been normalized by participant standard deviation for that task and metric.

*Denotes significance (

* *p < 0.05*,

** *p < 0.01*,

*** *p < 0.001)*.

**Figure 7:**
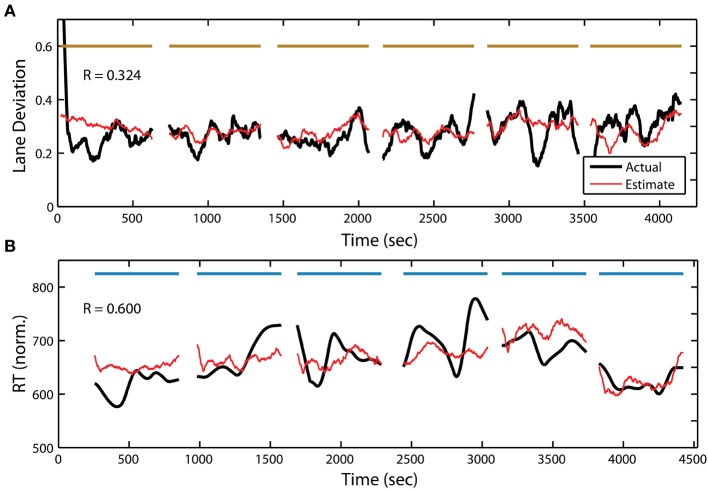
**Continuous measure of driving and RSVP (reaction time) behavior for participant S08. (A)** Actual and estimated absolute lane deviation (meters) over the 6 driving blocks. **(B)** Actual and estimated normalized RT (ms) over the 6 RSVP blocks. Horizontal bars indicate experiment blocks.

**Figure 8:**
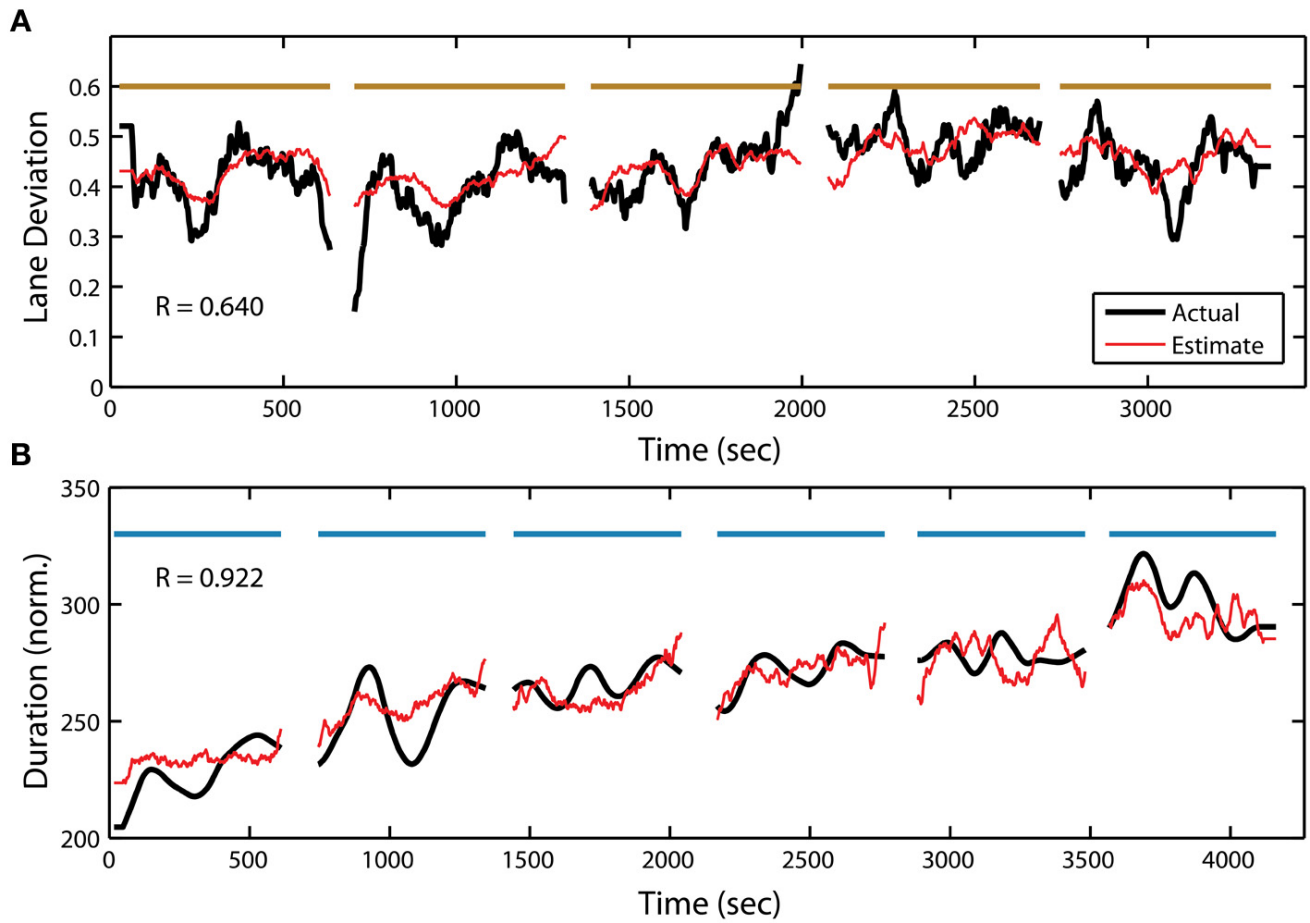
**Continuous measure of driving and RSVP (button press duration) behavior for participant S25. (A)** Actual and estimated absolute lane deviation (meters) over the 5 driving blocks. Note: last driving block not included due to data acquisition malfunction. **(B)** Actual and estimated normalized duration (ms) over the 6 RSVP blocks. Horizontal bars indicate experiment blocks.

In addition to target detection accuracy, we wanted to quantify the relationship between the PSD and the two other behavioral measures within the RSVP task. To accomplish this, we used the same adaptive modeling scheme described above to fit regression models and construct estimates for both normalized RT and button press duration. Figure 7B shows the actual and estimated RT for one participant in the RSVP task, while Figure 8B shows the actual and estimated button press duration for another participant. Here, estimates of lane deviation from their corresponding driving tasks are included for comparison (Figures 7A, 8A). Interestingly, our adaptive approach was able to produce significant behavioral estimates for both the RT and duration metrics in the majority of participants (RT *n* = 14, duration *n* = 17). The average correlation coefficients from these behavioral estimates were similar (RT *R* = 0.332 ± 0.225, duration *R* = 0.360 ± 0.302) to the normalized accuracy metric.

We observed no significant difference in the average correlation coefficient from the three RSVP metrics. Likewise, estimation accuracy from the three metrics was not significantly correlated across participants. This suggests that substantial individual differences exist in the link between the PSD and behavior in the RSVP task. For example, we were able to generate highly significant behavioral estimates along all three RSVP metrics for some participants (Table 4, participant 6). In other cases, only one of the three RSVP metrics produced a significant estimate (Table 4, participant 2). Across the population, the adaptive modeling scheme failed to produce a significant behavioral estimate for only 2 of the 25 participants in the RSVP task. Together, all participants had a least one significant estimate in either their driving or RSVP tasks.

### Topological and spectral features

**Figure 9:**
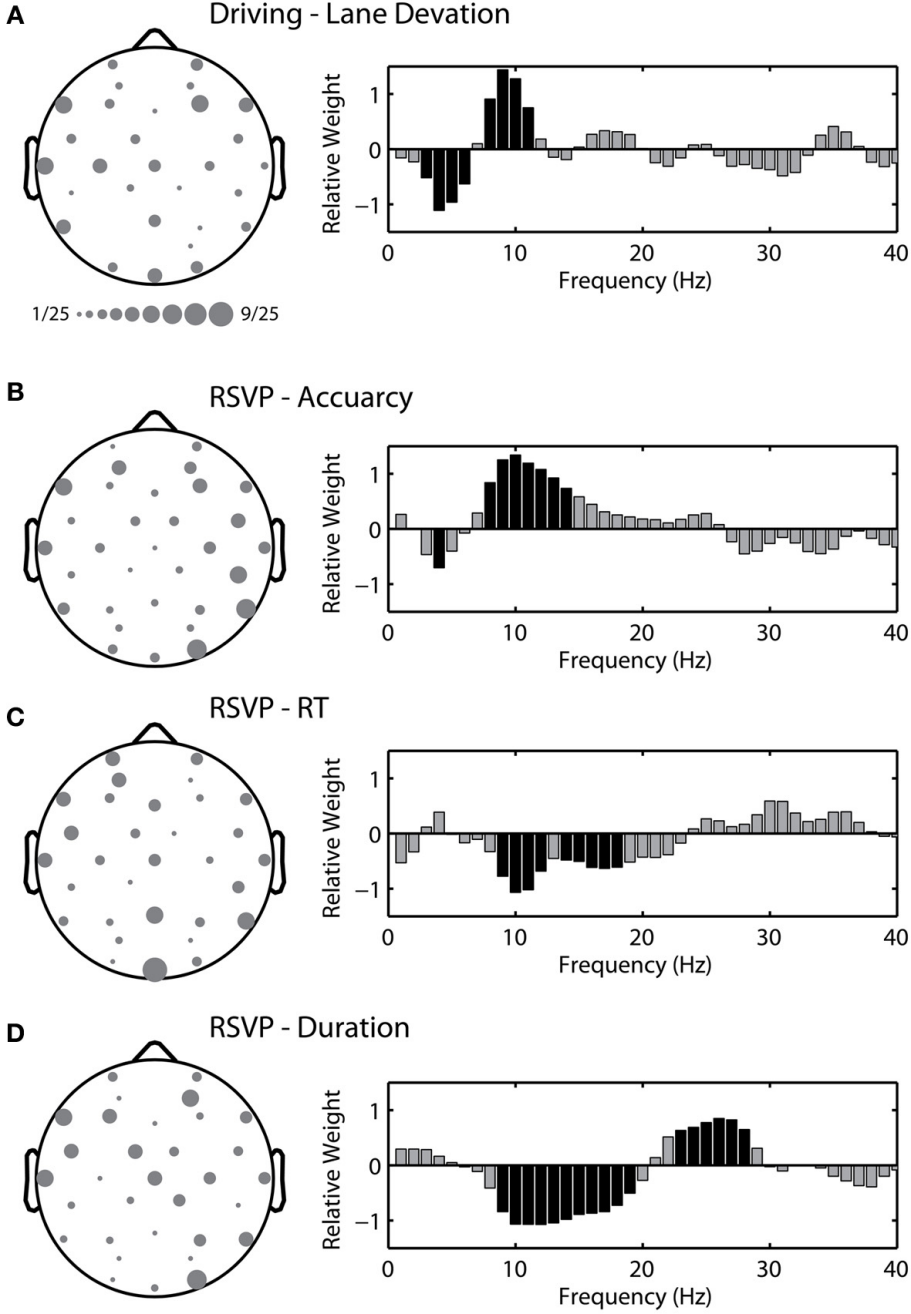
**Topological and spectral distribution of the adaptive model in the driving and RSVP tasks. (A)** Grand average channel montage (left) and spectrum of relative weights (right) for the optimal model in the driving task. Size of circle indicates relative frequency of inclusion across participants. Black shading indicates spectral weights with values significantly different than zero (*p* < 0.05 with FDR correction). **(B–D)** Channel montage and spectrum for the three behavioral metrics in the RSVP task.

Figure 9 shows the average topological distribution of included channels in the adaptive modeling scheme for both the driving and RSVP tasks. To quantify the gross features of this topology, we used the following approach. First, for each participant we normalized the channel distribution by the total number of channels included in their optimal model (between 1 and 12). We then separated the normalized distribution by hemisphere in two ways: anterior-posterior and left-right. For the first comparison we utilized the driving and RSVP-accuracy distributions. We performed an ANOVA with two factors (task × location) but did not identify any significant topological effects between tasks. We then performed an additional two factor ANOVA (metric × location) for the distributions within the RSVP task. While there was no significant clustering of channels across all metrics, there was a significant interaction between metric and left-right distribution [*F*_(2, 24)_ = 3.497, *p* < 0.05]. Here, the accuracy and duration models tended to select more channels from the right hemisphere.

In addition to the topological distributions, we wanted to quantify the spectral characteristics of the regression model. To accomplish this we calculated the relative weight for each frequency component within the linear model (1 to 40 Hz). Figure 9 shows the average spectrum of relative weights for both tasks. For the driving task, we found that models tended to include an inverse but balanced weighting of theta (4 to 7 Hz) and alpha (8 to 12 Hz) bands, very similar to previous reports (Lin et al., 2012). For the RSVP task, the accuracy and RT metrics exhibited a near-opposite spectral weighting, including components of both the alpha and beta (13 to 30 Hz) bands. This likely reflected their complementary relationship to time-on-task changes in performance (i.e., decreases in accuracy and increases in RT over time). Interestingly, the spectral weights for the duration metric included both positive and negative values within the beta band.

The topological distributions show some level of commonality in the electrodes selected by the adaptive modeling scheme for both the driving and RSVP tasks. The most commonly selected electrode was Oz for models estimating RT in the RSVP task (Figure 9C). We wanted to determine the relative influence of the most commonly selected channels in each task. To do this we built fixed-montage models using the four most commonly selected electrodes in each task and for all three RSVP metrics (i.e., a different fixed-montage for each condition). We found that the performance of these models was very similar to the standard modeling scheme (utilizing Fz, Cz, Pz, and Oz). For the driving task, the models using the four most commonly selected electrodes produced an average correlation coefficient of 0.081 ± 0.279, similar to the standard scheme (*R* = 0.079 ± 0.224). For the RSVP task, the average correlation coefficients were similar for accuracy (common electrodes: *R* = −0.051 ± 0.347, standard scheme: *R* = −0.137 ± 0.332), RT (common electrodes: *R* = −0.004 ± 0.292, standard scheme: *R* = 0.031 ± 0.266), and duration (common electrodes: *R* = −0.034 ± 0.388, standard scheme: *R* = −0.073 ± 0.388). These results suggest that the power of the adaptive modeling scheme is in the ability to capture the large, inter-subject variability in both electrode number and location.

### Model generalization

**Figure 10:**
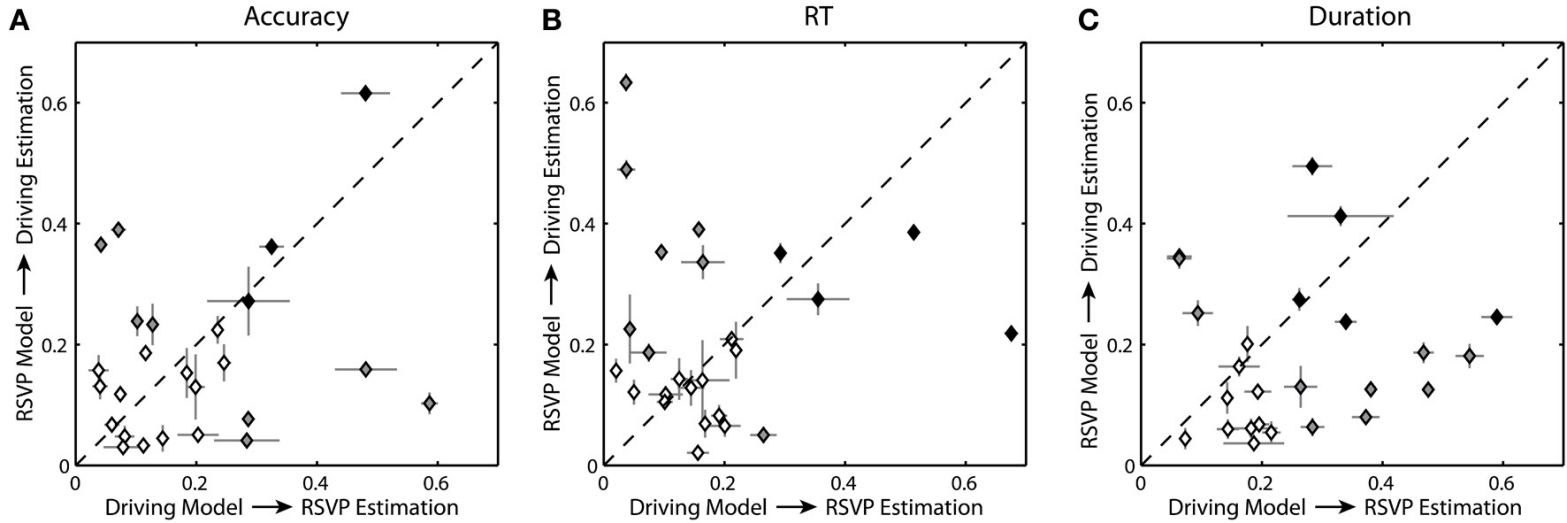
**Cross-task estimation accuracy. (A)** Average correlation coefficient between the cross-task estimated and actual behavior for each participant using normalized accuracy as the RSVP behavioral metric. **(B,C)** Average correlation coefficients using normalized RT and duration respectively. Level of shading indicates significance: black = significant for both conditions (*p* < 0.05), gray = significant for one condition (*p* < 0.05), clear = not significant. Horizontal and vertical bars show the standard error for the distribution of correlation coefficients.

The regression models described above are optimized for each participant and task. However, some elements of these models may have the ability to generalize across tasks. In particular, one of the three behavioral metrics in the RSVP task may produce more generalizable models than the others. To explore this, we used the regression models from the driving task to estimate RSVP behavior and models from the RSVP task to estimate driving behavior. For each participant, we used the six models from the cross-validation process and applied them to the entire PSD data from the alternate task, resulting in six complete estimates of the behavior for each participant and task. This process was repeated for each of the behavioral metrics in the RSVP task: normalized target detection accuracy, RT, and button press duration. Importantly, we needed to scale the estimate to the new behavioral metric. For driving, the metric (absolute lane deviation) typically increases with time-on-task fatigue or boredom; in contrast, the RSVP metric (target detection accuracy) typically decreases under the same conditions. Thus, we added an additional linear transform, slope, and offset, to match the novel behavioral metric. While this additional transform corrects for the sign and scale of the linear relationship it does not affect the magnitude of the correlation coefficient (i.e., the measure of estimation accuracy). Figure 10 shows the distribution of these cross-task correlation coefficients (mean and standard error) for all participants.

Across participants, regression models constructed from the driving data were able to estimate some degree of the behavioral variation in the RSVP task, and vice versa. Specifically, for the regression models built on the driving task the average correlation coefficient between the actual and estimated RSVP accuracy was 0.195 ± 0.150. Only 7 of the 25 participants had significant correlation coefficients. For the RSVP task models based on accuracy, the average correlation coefficient between the actual and estimated lane deviation was 0.176 ± 0.140. In this instance a different subset of participants (7 of the 25) had significant correlation coefficients. For RT and duration, the results were similar. The average correlation coefficient between the actual estimated RSVP behavior was 0.183 ± 0.152 (5/25 significant) for RT and 0.259 ± 0.148 for duration (12/25 significant). Estimates of lane deviation yielded average correlation coefficient of 0.218 ± 0.151 (11/25 significant) for RT-based models and 0.177 ± 0.123 (8/25 significant) for duration-based models. As the scatter plots suggest (Figure 10), we did not observe a significant correlation between the accuracy of the behavioral estimates within these two tasks.

## Discussion

In this paper, we have extended an approach for modeling elements of instantaneous driver performance based on changes in the EEG log power spectra, and we have evaluated this approach by estimating continuous performance in a simulated driving task. We were able to generalize this approach to estimate fluctuations in RSVP behavior (target detection accuracy, RT, and button press duration) to a similar degree. Furthermore, when regression models fit under the driving paradigm were applied to the RSVP task, explained variance remained significant for some participants, despite its reduction overall. Together, our results show the potential for estimating time-on-task performance decrements in current and future BCI-relevant paradigms. While average accuracy of the estimated driver performance was lower than previous reports (Lin et al., 2005a,b), our results are from a larger cohort of participants that were not particularly fatigued at the time of the experiment. In addition, our driving simulator incorporated more complex vehicle dynamics, including operator control over vehicle speed, which further extended the realism of this study.

It is important to note that the major source of behavioral variance in our RSVP paradigm was not time-on-task. Despite strong indicators of increasing subjective fatigue and boredom, target class was a primary modulator of performance across blocks (Figures 4, 5). This phenomenon was due to the difference in the average perceptual difficulty in the identification of objects from each target class. While target images from each class were roughly matched along low-level visual dimensions such as luminance, object size, and eccentricity, we observed a significant effect of target class in all three behavioral metrics (target detection accuracy, RT and button press duration). Likewise, we observed a similarly strong effect of target probability on both RT and duration. Once we normalized the behavioral metrics for these factors, a clear time-on-task effect was evident in target detection accuracy. While RT and duration did not show the same effect at the block level, significant linear trends were observed within experimental blocks. Interestingly, all three behavioral metrics were able to produce models with similar explanatory power.

The adaptive modeling scheme produced topological distributions (Figure 9) that reflect elements of the underlying neural processes. The driving and RSVP tasks likely engaged a range of brain networks with some degree of overlap between tasks. The majority of work exploring the link between neural activity and driver performance has focused on the central to occipital regions (Borghini et al., 2012; Lin et al., 2012). In contrast, the majority of RSVP target classification studies have implicated frontal-parietal networks (Gerson et al., 2005; Luo and Sajda, 2009). Our results indicated that a broad range of channels were selected in both the driving and RSVP tasks. However, since the adaptive modeling scheme was a primarily data-driving approach to behavior estimation, inferences regarding the foci of underlying network activity are limited. In general, the number of channels included in each participant's optimal model was small (driving = 3.72, RSVP accuracy = 4.32, RSVP RT = 4.24, RSVP duration = 4.08). SFFS ranks the channels based on cumulative predictive power, thereby minimizing redundancy in the feature selection process. Hence, the lowest ranking (most significant) channels tend not to be spatially adjacent, obscuring the underlying scalp distribution. In addition, there was substantial variability in both the number and location of selected channels, making average topological distributions difficult to interpret, even with a relatively large sample size (*N* = 25).

In contrast to the topology, the spectral features showed some commonality across participants. Every linear model contained a set of weights associated with each eigenvector, which in turn represents a combination of spectral features. By calculating the relative weight for each frequency across all participants, we were able to assess the spectral features associated with the behavior in each task. Not surprisingly, the models for the driving task incorporated the theta and alpha bands in their estimation of lane deviation. This relative combination of theta and alpha was very similar to previous reports linking neural activity to changes in driver performance (Lin et al., 2010b, 2012; Chuang et al., 2012). For the RSVP task, the spectral features were dependent on the behavior of interest. Target detection accuracy was found to be positively associated with frequencies in the alpha band. This result is in contrast to previous research which has shown that visual discrimination performance is negatively associated with alpha power during pre-stimulus periods, primarily in the bilateral parieto-occipital regions (Thut et al., 2006; Hanslmayr et al., 2007; Romei et al., 2008; Van Dijk et al., 2008; Mathewson et al., 2009, 2011). However, these studies support the notion that pre-stimulus alpha activity can function as an attentional gating mechanism by which task-irrelevant information is inhibited (Jensen and Mazaheri, 2010; Foxe and Snyder, 2011). Thus, the observed positive relation between alpha power and accuracy may be a reflection of inhibitory responses to the relatively more frequent non-target stimuli.

The spectral profile of the RT models exhibited a near-opposite weighting compared with models of accuracy. This is consistent with the observed behavior, where RT and accuracy showed a complementary relationship to time-on-task changes in performance. Again, our results are in contrast to previous research relating pre-stimulus alpha to decreased accuracy and increased RT. However, there is a clear difference between pre-stimulus measures of spectral power and PSD estimates calculated during the RSVP (Macdonald et al., 2011). The steady state visually evoked potential (SSVEP) has a strong influence on the spectral profile and produces peaks at the presentation frequency (in this case 5 Hz) and corresponding harmonics. In addition, the SSVEP itself is modulated by the attentional state of the participant (Kim et al., 2007). Thus, the spectral features contained within the models of RSVP behavior likely reflect an interaction between the SSVEP and endogenous changes in oscillatory activity.

For button duration, a negative relationship was observed in the alpha and low beta bands while a positive relationship was observed in the high beta band. This finding appears consistent with the event-related alpha desynchronization/synchronization (ERD/ERS) and post-movement beta synchronization literature. Previous research has consistently shown alpha (10 to 12 Hz mu rhythm) and a harmonic beta (20 to 24 Hz) desynchronization preceding finger movements, as well as post-movement beta synchronization in the 12 to 16 Hz and 26 to 30 Hz bands (Pfurtscheller and Lopes da Silva, 1999). However, as with accuracy and RT, these results may reflect a complex interaction between stimulus- and response-related activities. Further experimentation is required in order to differentiate the neural and functional sources of the spectral features contained within our behavior estimation models.

Given the nature of our adaptive modeling scheme, exploring the link between the topological and spectral distributions would be a substantial challenge. The relative spectral weights are extracted from the eigenvector loadings. The spatially distributed channels that constitute each model are therefore spectrally linked through the PCA process. Thus, determining the independent influence of PSD spectra at each spatial location would be difficult. In contrast, alternative approaches often have an initial, separate feature selection process. For example, other groups have used independent component analysis (ICA) to first identify a small number of components with unique scalp topologies that are maximally correlated with the behavior of interest (Lin et al., 2012). Similarly, other groups first calculate the average power within established frequency bands: most notably delta, theta, and alpha (Balasubramanian et al., 2011). While substantially limiting the dimensionality of the feature space, these initial selection processes allow for a more direct association between spatial or spectral features and changes in behavior.

### Linear model considerations

Interestingly, there was substantial variability in model performance across blocks (Figure 6). This may have resulted from several factors. First, the relationship between the neural signature of fatigue and the resulting behavior may have itself changed over time. During the course of the task, participants could have engaged in different compensatory strategies for perceived time-on-task fatigue (Hockey, 1997) and these strategies could have differentially affected the observed behavior. This was especially true for the RSVP task in which there was a tradeoff between the speed and accuracy of the response. Second, the observed increase in perceived boredom over each task could have negatively influenced the motivation of participants to mitigate fatigue induced performance decrements. For example, the time it takes to return the vehicle to the cruising lane after the onset of a perturbation (i.e., response time) could have been negatively affected by motivation. In turn, this would have had a large impact on the smoothed lane deviation values with only an indirect link to time-on-task fatigue. Finally, while our PSD estimation process utilized band-pass filters and the power spectra were smoothed over a 90 s integration window, external noise and muscle artifacts could have degraded the model fitting process. Indeed, ICA has been used in similar approaches to mitigate artifacts and improve the signal-to-noise ratio (Lin et al., 2005b, 2006, 2012).

While the current study employed a data-driven approach designed to estimate changes in observed behavior, we sought to constrain the dimensionality of the problem and leverage previous work within this area. Several studies have identified the general time course of behavioral fluctuation from endogenous sources such as fatigue or alertness level (Jung et al., 1997; Lin et al., 2005b; Chuang et al., 2012). Our 90 s mean filter was directly borrowed from this previous work. However, this parameter imposes a constraint on the distribution of random correlation coefficients. Low-pass filtered random signals can achieve spurious correlations of relatively high magnitude. While a broader temporal integration window would likely increase the average accuracy, it would equivalently increase the significance threshold. Thus, given that our results predominately captured slow changes in performance (e.g., across blocks), our integration window was well suited to the nature of the behavioral fluctuations we attempted to estimate.

The analytical approach described here employs a relatively simple model, combined with feature selection, to generate continuous estimates of behavior. Similar approaches have been extended to incorporate ICA (Lin et al., 2005b) and fuzzy neural-networks (Lin et al., 2006, 2012). However, the linear approach still represents a solid and interpretable framework for exploring the relationship between the EEG power spectra and behavior in a variety of tasks. In addition, this method is computationally simple and utilizes universal signal processing components such as continuous PSD estimation from channel data. Thus, as we have demonstrated here, it remains a practical approach for an embedded application in current BCI systems (Lin et al., 2010a).

A number of other approaches for predicting changes in performance, due to fatigue or workload, have targeted specific frequency bands within the PSD (e.g., alpha and theta). As described above, the benefit of this more directed approach is the substantial reduction in the dimensionality of the feature space. In turn, this allows for the creation of more robust models with less data and a tractable exploration of frequency band interactions (e.g., theta-alpha ratio). However, *a priori* selection of features can limit a model's explanatory power by averaging over or ignoring PSD features that potentially contain information. The method utilized here, in contrast, takes a data-driven approach to feature selection, both in the channel montage and PSD components. While this more flexible approach is ideal for catching variations across individuals, it may be constrained in its ability to estimate behavior in novel tasks.

### Increasing BCI robustness

As BCI technologies improve, one potential application of this approach is as an opportunistic tool (Lance et al., 2012) within current and future applications, such as an image triage BCI system (Gerson et al., 2006; Sajda et al., 2010). While driver performance estimation systems that seek to use EEG must significantly outperform other approaches and modalities in order to justify the imposition of EEG signal acquisition, active and reactive BCI systems already include real-time neural signal processing. Thus, the addition of time-on-task behavior estimation algorithms would opportunistically take advantage of that data at little or no additional cost. The results of this study provide an initial proof-of-concept showing that existing fatigue-based performance estimation algorithms can be repurposed for additional BCI-specific tasks. This BCI-within-a-BCI framework potentially opens several future possibilities, most notably by using the performance estimation algorithm to increase the robustness of extended use active or reactive BCI technologies through a variety of mitigation methods (Zander and Kothe, 2011; Zander and Jatzev, 2012).

There are many possible approaches to developing these mitigation methods. One is to have different target-detection classifiers optimized to match individual fatigue levels. Another is to use the performance estimation algorithm to adapt the RSVP task to the user's current performance level; for example by re-presenting images seen during periods of low performance or simply slowing the presentation rate. It should also be possible to affect the interaction of an RSVP BCI with a computer vision (CV) system, such as the system described in Gerson et al. (2006) or Touryan et al. (2013b); for example by weighting the target labels provided by the CV system more highly when the user is in a period of low performance. A related hybrid approach, combining measures of covert spatial attention with a motor imagery BCI, was recently proposed by Tonin et al. (2013).

## Conclusion and future work

Typically, EEG-based performance estimation algorithms, like the one described here, are designed for tracking slow fluctuations in behavior. These approaches are intended to provide an objective measure of changes in task-induced fatigue (Lin et al., 2012) or mental workload (Kohlmorgen et al., 2007; Brouwer et al., 2012) that directly affect behavior over longer periods of time. In this study, we adapted one such approach and extended its application into a novel task paradigm. Our results show the potential for using performance estimation algorithms to inform current and future BCI applications by addressing some of the non-stationarities inherent within neural signals. By identifying time-on-task performance decrements, this approach could ultimately lead to more robust BCI systems.

Unfortunately, while our adaptive approach produced significant behavioral estimates in each task, a challenge remains in developing a universal, task-independent model of performance decrements. The adaptive modeling scheme described here is optimized for a particular individual, task, and behavior. However, brain activity clearly varies between individuals, across tasks and over time. Thus, behavioral models based on a particular ensemble of EEG data tend to degrade in their ability to extrapolate across these factors. However, there are analysis methods such as transfer learning (Pan and Yang, 2010), that may improve this extrapolation process. These techniques have recently been applied to BCIs (Lu et al., 2009; Jin et al., 2013; Samek et al., 2013; Wu et al., 2013), and provide a potential next step for extending this work.

### Conflict of interest statement

The authors declare that the research was conducted in the absence of any commercial or financial relationships that could be construed as a potential conflict of interest.
