# Supplementary material for: Estimating endogenous changes in task performance from EEG
Source: Front Neurosci. 2014 Jun 13;8:155. doi: 10.3389/fnins.2014.00155 (PMC4061490; doi:10.3389/fnins.2014.00155)
Supplement: Supplementary file 1 [file DataSheet1.DOCX]

***Supplementary Material***

**Estimating Endogenous Changes in Task Performance from EEG**

**Jon Touryan*, Gregory Apker, Brent J. Lance, Scott E. Kerick, Anthony J. Ries, and Kaleb McDowell**

Human Research and Engineering Directorate, U.S. Army Research Laboratory, Aberdeen Proving Ground, MD 21005, USA

*** Correspondence:** Dr. Jon Touryan, U.S. Army Research Laboratory, ARL/HRED, RDRL-HRS-C, Aberdeen Proving Ground, MD 21005, USA; e-mail: [jonathan.o.touryan.ctr@mail.mil](mailto:jonathan.o.touryan.ctr@mail.mil)

1. **Cross-validation Procedure**

The leave-one-out cross-validation approach described in this study was adapted to the experimental paradigm (6 blocks, 10 minutes in length). Regression models were constructed on data from five blocks and tested on the remaining block. The benefit of this approach was that there existed a natural break between blocks, typically lasting more than a minute. These breaks insured a statistical separation between training and testing sets. Specifically, the inclusion of a 90 second (mean filter) smoothing window imposed a statistical relationship between adjacent samples within that timeframe. Thus, it was critical to separate the training and testing sets by a temporal gap of at least this size. Unfortunately, this restricted the potential k-fold cross-validation schemes. However, it remained important to quantify the relationship between the cross-validation approach, specifically the validation window size, and the model performance.

To quantify how estimation accuracy was affected by the validation window size, or number of folds in the k-fold validation scheme, we performed the following analysis. We fit regression models, using the adaptive approach, under a range of window sizes. The sizes were chosen to span a reasonable range of values given the length of each task (60 minutes) and the size of the smoothing window (1.5 minutes). We consistently enforced a 90 second separation between the training and testing sets at each fold, excluding training data adjacent to the testing set. **Supplementary Figure 1** shows the average model performance, across all participants, as a function of validation window size. To determine if there was any statistical difference in the average performance we use an ANOVA with window size as the factor. We found that the effect of window size on average performance was not significant in either the driving (*F*(4,24) = 0.10, *p* = 0.41) or RSVP (*F*(4,24) = 1.31, *p* = 0.27) tasks.


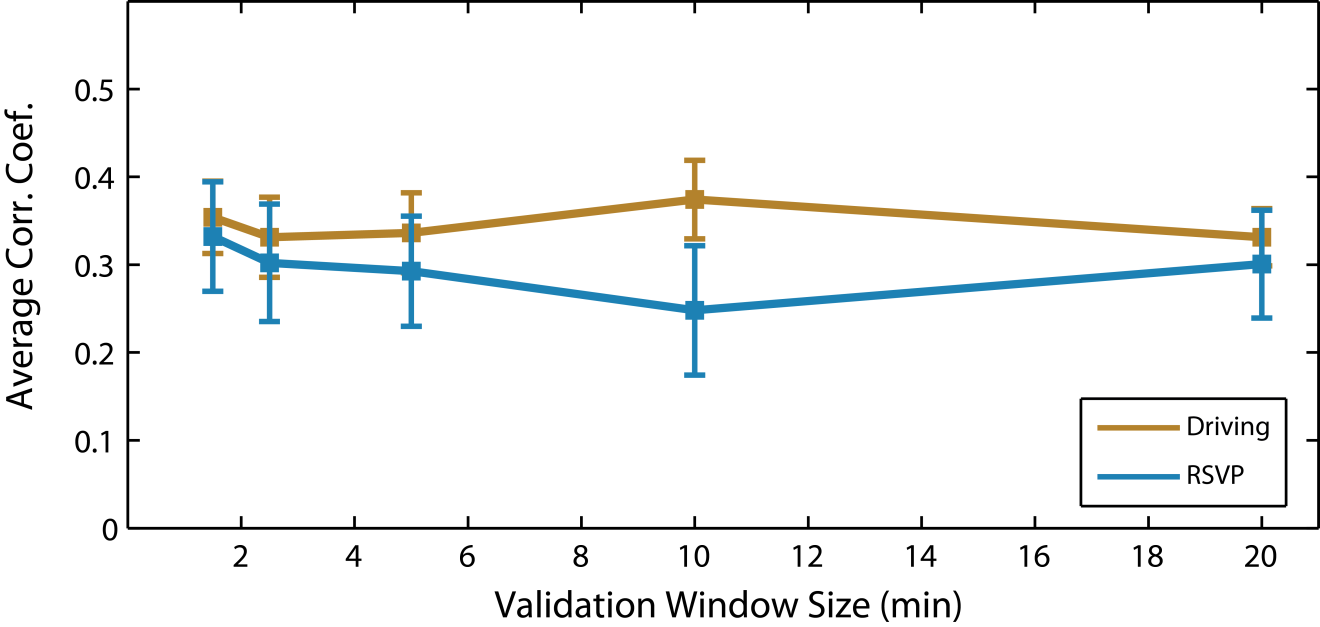


**Supplementary Figure 1:** Grand average correlation coefficient as a function of validation window size. The behavioral metric in the RSVP task was accuracy. A validation window size of 10 minutes matched the length of the experimental blocks.

Noticeably, there was a divergence in r-values when using a 10 minute validation window. This was most probably due to alignment with the experimental blocks. As shown in the example estimates (**Figures 6-8**) there was often a small shift in the baseline performance across blocks. Between blocks participants were responding to surveys and often returned to the task at a slightly different baseline performance level. This cross-block shift tended to improve the estimates of driving behavior but degraded the estimates for RSVP behavior. The RSVP specific reduction may be due to task changes between blocks (i.e., new target class and new target frequency). Using the 10 minute validation window, as compared to a 5 minute window, did result in a significant increase in accuracy for the driving task (*p* = 0.02; paired t-test) but not the RSVP task (*p* = 0.25). However, the cross-validation ANOVA (described above) did not identify validation window size as a significant factor for either the driving or RSVP tasks.

**Supplementary Figure 2** shows the actual and estimated behavior from one participant with three different k-fold validation schemes. While estimation accuracy varies between the validation schemes, the estimated behavior does not qualitatively change as the window size shrinks. Likewise, **Supplementary Table 1** shows the combined model performance using a 5 minute validation window. The table contains data from all participants in each task, including the three behavioral metrics of the RSVP task. The RMSE values and correlation coefficients fluctuate across participants (compared to **Table IV**). Likewise, a few models change their level of significance (in both directions). However, there is no systematic improvement in model performance compared with the 10 minute validation window.


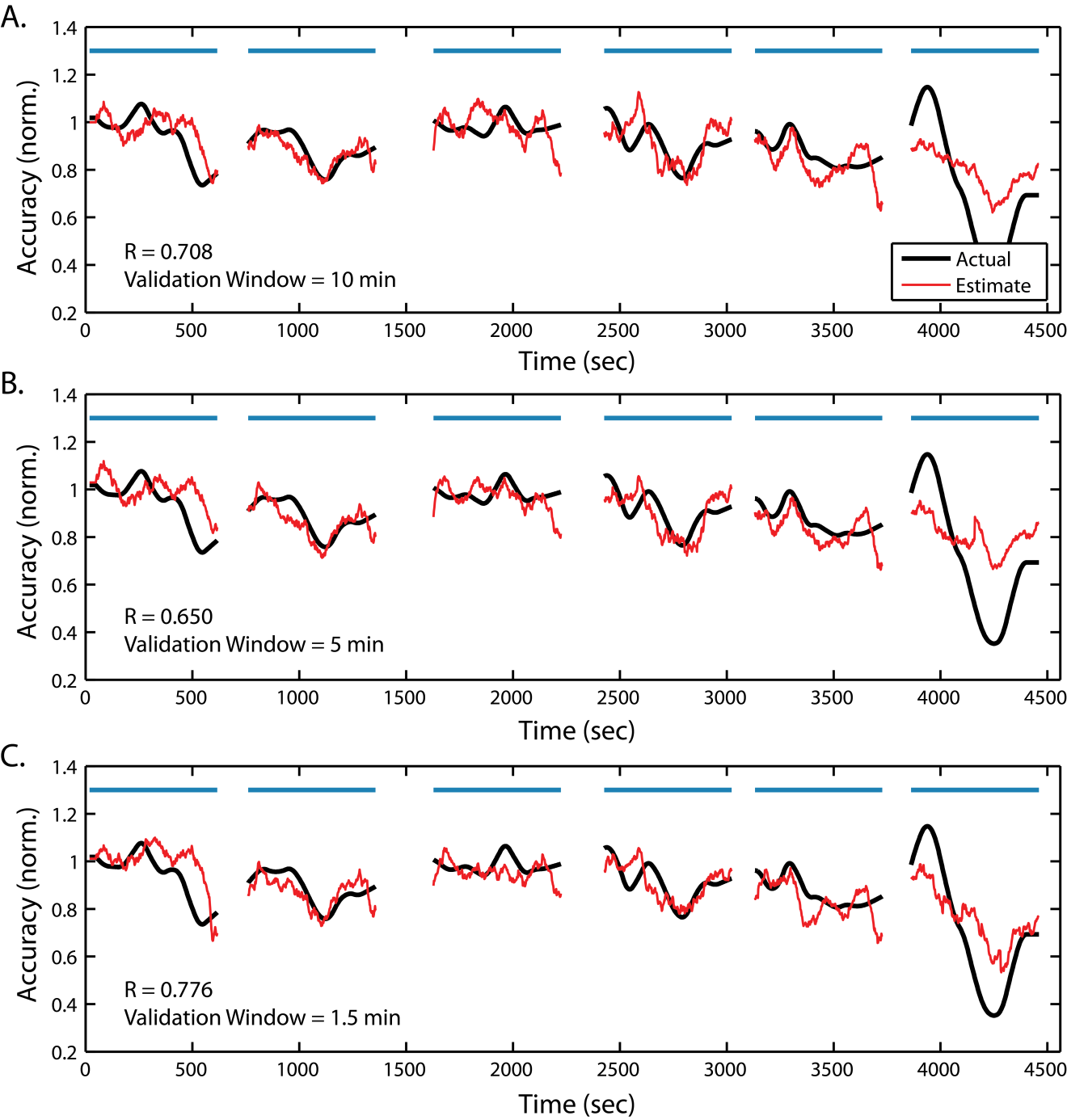


**Supplementary Figure 2:** Continuous measure of RSVP behavior for participant S22. A) Actual and estimated accuracy over the 6 RSVP blocks using the standard 10 min validation window size. B) Actual and estimated accuracy using a 5 minute validation window size. C) Actual and estimated accuracy using a 1.5 minute validation window size. Horizontal bars indicate experiment blocks.

**Supplementary Table 1**

Regression model performance for the driving and RSVP tasks using 5 min cross-validation window

|  | **Driving** | | | **RSVP-Accuracy** | | | **RSVP-RT** | | | **RSVP-Duration** | | |
| --- | --- | --- | --- | --- | --- | --- | --- | --- | --- | --- | --- | --- |
| **Participant** | **RMSE^1^** | **R** |  | **RMSE^1^** | **R** |  | **RMSE^1^** | **R** |  | **RMSE^1^** | **R** |  |
| 1 | 0.81 | 0.58 | *** | 0.93 | 0.39 | ** | 0.93 | 0.40 | ** | 0.97 | 0.30 | * |
| 2 | 0.96 | 0.30 | ** | 0.92 | 0.44 | ** | 1.13 | -0.13 |  | 1.19 | -0.22 |  |
| 3 | 0.88 | 0.49 | *** | 0.95 | 0.38 | ** | 1.01 | 0.16 |  | 0.93 | 0.38 | ** |
| 4 | 1.17 | -0.21 |  | 0.82 | 0.57 | *** | 0.81 | 0.59 | *** | 0.82 | 0.58 | *** |
| 5 | 0.98 | 0.30 | ** | 1.02 | 0.15 |  | 0.90 | 0.46 | *** | 1.00 | 0.27 | * |
| 6 | 1.03 | 0.06 |  | 0.64 | 0.77 | *** | 0.82 | 0.57 | *** | 0.88 | 0.48 | *** |
| 7 | 1.01 | 0.29 | ** | 1.17 | -0.35 |  | 1.02 | 0.17 |  | 1.07 | -0.09 |  |
| 8 | 0.96 | 0.32 | *** | 1.08 | 0.09 |  | 0.81 | 0.59 | *** | 1.09 | -0.02 |  |
| 9 | 1.02 | 0.19 |  | 1.05 | 0.14 |  | 1.03 | 0.04 |  | 1.02 | 0.17 |  |
| 10 | 0.83 | 0.57 | *** | 0.97 | 0.38 | ** | 1.05 | 0.11 |  | 1.17 | -0.37 |  |
| 11 | 0.83 | 0.56 | *** | 0.68 | 0.73 | *** | 1.00 | 0.22 |  | 0.75 | 0.66 | *** |
| 12 | 0.98 | 0.27 | * | 0.97 | 0.26 | * | 0.89 | 0.46 | *** | 0.86 | 0.51 | *** |
| 13 | 1.11 | -0.29 |  | 1.00 | 0.20 |  | 0.81 | 0.59 | *** | 0.75 | 0.67 | *** |
| 14 | 0.90 | 0.45 | *** | 1.14 | -0.02 |  | 0.92 | 0.39 | ** | 0.90 | 0.44 | ** |
| 15 | 0.91 | 0.42 | *** | 0.83 | 0.56 | *** | 1.00 | 0.21 |  | 0.87 | 0.51 | *** |
| 16 | 0.96 | 0.34 | ** | 0.76 | 0.65 | *** | 0.69 | 0.73 | *** | 0.96 | 0.33 | ** |
| 17 | 0.91 | 0.43 | *** | 1.07 | 0.05 |  | 0.80 | 0.60 | *** | 0.78 | 0.64 | *** |
| 18 | 1.00 | 0.29 | ** | 0.92 | 0.42 | ** | 0.99 | 0.27 | * | 0.63 | 0.77 | *** |
| 19 | 0.85 | 0.55 | *** | 1.07 | 0.15 |  | 0.82 | 0.58 | *** | 1.12 | 0.10 |  |
| 20 | 0.77 | 0.64 | *** | 0.76 | 0.65 | *** | 1.05 | 0.20 |  | 0.83 | 0.58 | *** |
| 21 | 0.96 | 0.36 | ** | 0.81 | 0.60 | *** | 1.02 | 0.23 |  | 0.70 | 0.71 | *** |
| 22 | 0.81 | 0.59 | *** | 0.99 | 0.24 |  | 0.89 | 0.49 | *** | 0.40 | 0.92 | *** |
| 23 | 0.98 | 0.26 | * | 0.94 | 0.39 | ** | 0.74 | 0.67 | *** | 0.76 | 0.65 | *** |
| 24 | 0.92 | 0.41 | *** | 1.11 | -0.05 |  | 1.05 | 0.19 |  | 1.15 | -0.16 |  |
| 25 | 0.99 | 0.23 | * | 1.21 | -0.46 |  | 1.02 | 0.20 |  | 0.86 | 0.54 | *** |
|  |  |  |  |  |  |  |  |  |  |  |  |  |
| **Average** | **0.94** | **0.34** |  | **0.95** | **0.29** |  | **0.93** | **0.36** | **0.93** | **0.90** | **0.37** |  |

^1^ RMSE values have been normalized by participant standard deviation for that task and metric.

* Denotes significance (* *p* < 0.05, ** *p* < 0.01, *** *p* < 0.001)
